# Jasmonate resistant 1 and ethylene responsive factor 11 are involved in chilling sensitivity in pepper fruit (*Capsicum annuum* L.)

**DOI:** 10.1038/s41598-022-07268-3

**Published:** 2022-02-24

**Authors:** Jeong Gu Lee, Jieun Seo, Byoung-Cheorl Kang, Jeong Hee Choi, Eun Jin Lee

**Affiliations:** 1 Department of Agriculture, Forestry and Bioresources, College of Agriculture and Life Sciences, Seoul National University, Seoul, 08826 Republic of Korea; 2 Plant Genomics and Breeding Institute, Seoul National University, Seoul, 08826 Republic of Korea; 3 Korea Food Research Institute, Wanju-gun, Jeollabuk-do 55365 Republic of Korea; 4 Research Institute of Agriculture and Life Sciences, Seoul National University, Seoul, 08826 Republic of Korea

**Keywords:** Physiology, Plant sciences

## Abstract

Pepper fruit (*Capsicum annuum* L.) is sensitive to chilling stress with chilling injuries occurring below 7 °C; however, chilling injuries occur at different temperatures depending on the genotype. The present study aimed to identify the factors that affect chilling sensitivity in pepper fruits. A total of 112 F_2_ pepper fruits crossed between chilling-insensitive '*UZB-GJG-1999–51*' and chilling-sensitive '*C00562*' pepper were grouped according to the seed browning rate, which is a typical chilling symptom of pepper fruit under chilling conditions. Physiological traits, amino acids, fatty acids, as well as *ethylene responsive factor* (*ERF*) and *jasmonate resistant 1* (*JAR1*) expression levels were analyzed, and their correlations with the seed browning rate were confirmed. The expression level of *JAR1* showed a strong negative correlation with the seed browning rate (r =  − 0.7996). The expression level of *ERF11* and content of hydrogen peroxide showed strong positive correlation with the seed browning rate (r = 0.7622 and 0.6607, respectively). From these results, we inferred that *JAR1* and *ERF11* are important factors influencing the chilling sensitivity of pepper fruit.

## Introduction

Pepper (*Capsicum annuum* L.) is a subtropical vegetable that is sensitive to chilling stress. Thus, when subjected to chilling stress below 7 °C, various symptoms of chilling injury appear depending on the genotype, ripening stage, and treatment period of chilling^1^. In pepper fruit, the most common symptom of chilling injury is seed browning, which leads to reduced quality and economic loss after harvest. One of the main causes of seed browning is enzymatic browning by phenolic compounds. Total phenol content is associated to the oxidative browning of pepper seeds, and it has been reported that seed browning, induced by chilling stress, is related to polyphenol oxidase (PPO) and phenylalanine ammonia lyase (PAL)^2^. Phenolic compounds are biosynthesized from phenylalanine, the precursor of phenolic compounds, by PAL, and the generated phenolic compounds are then oxidized to quinone by PPO. Finally, a brownish pigment is formed, and tissue browning occurs^3^.

Other major causes of seed browning are membrane peroxidation and increased reactive oxygen species (ROS), such as hydrogen peroxide, in embryos^4^. Under chilling stress conditions, cell membranes undergo enzymatic or non-enzymatic peroxidation in which unsaturated fatty acids are oxidized to saturated fatty acids, resulting in ROS production^5^. The generated ROS, in turn, react with the cell membrane to promote cell membrane peroxidation. To suppress these cycles, jasmonic acid (JA) signaling is activated, regulating the concentration of metabolites, such as amino acids and sugars, or inhibiting ROS production through the regulation of genes involved in ROS scavenging.

JA is one of the plant hormones, synthesized from α-linolenic acid, which regulates plant growth and development extensively. In addition, JA plays an important role in response to herbivorous insects and to environmental stress^6^, also is involved in chilling response enhancing chilling tolerance^7,8^. In addition, JA accumulation under chilling stress has been reported in *Arabidopsis*^9^, tomatoes^10^, and pepper^11^. JA should be conjugated with isoleucine by jasmonate resistant 1 (JAR1) to form jasmonoyl-isoleucine (JA-Ile)^12,13^. Then the generated JA-Ile binds to CORONATINE INSENSITIVE 1 to promote the ubiquitination of the JASMONATE-ZIM-domain protein, which acts as a repressor of JA signaling^14^.

Representative genes regulated by JA signaling are in the ethylene responsive factor (ERF) family. The ERF family contains AP2/ERF-type binding domains and is composed of the ERF and dehydration responsive element binding factor (DREB) subfamily^15^. The ERF family is involved in abiotic stress in a variety of plants, and the ERF subfamily, including ERFs 1, 2, 5, 6, and 11, is mainly involved in osmotic stress^16^. In contrast, the DREB subfamily is known to be involved in chilling stress response in *Arabidopsis*^17,18^. In pepper, CaPTI1 belonging to the ERF subfamily is involved in cold and drought stress and in *Phytophthora capsici* infection^19^.

However, previous research on chilling stress response in peppers is insufficient because the response and sensitivity to chilling stress differ depending on the pepper genotype. In our previous study, we confirmed the factors affecting the chilling sensitivity of pepper fruits^20^. When the chilling-insensitive '*UZB-GJG-1999–51*' and chilling-sensitive '*C00562*' peppers were exposed to chilling treatment at 2 °C for 24 h, the expression levels of *CaJAR1*, *CaERF 1*, *3*, *5*, and *10* increased in both the genotypes. It was also confirmed that the expression levels of *CaERF11* and *CaDERB3* decreased in both the genotypes. In addition, the expression levels of *CaJAR1*, *CaERF 1*, *3*, *5*, and *10* were higher in chilling-insensitive '*UZB-GJG-1999–51*' pepper, and those of *CaERF11* and *CaDREB3* were higher in chilling-sensitive '*C00562*' pepper. As mentioned previously, *JAR1* is a major activator of JA signaling through JA-Ile synthesis^12,13^. In addition, *ERF 1*, *3*, and *5* were reported to be upregulated under chilling stress in *Arabidopsis*^21^ and cotton^22^, and *ERF10* has been shown to improve chilling tolerance in bananas^23^. *ERF11* is known to promote plant internode elongation by activating the synthesis of gibberellin. In general, under chilling stress, plants respond through growth retardation^24,25^. Therefore, *ERF11* seems to have a function of weakening the chilling tolerance. On this basis, in the chilling stress response, each of the former genes was expected to be a positive regulator, and the latter genes were expected to be candidate negative regulator genes.

In the present study, we investigated the factors that influence chilling sensitivity and seed browning in various genotypes of pepper fruit by confirming the correlations between the aforementioned candidate genes, ROS content, fatty acids, amino acids, and seed browning rate in pepper fruit.

## Material and methods

### Plant materials

We obtained pepper seeds of chilling-insensitive ‘*UZB-GJG-1999–51*’ and chilling-sensitive ‘*C00562*’ from Plant Genomics and Breeding Institute (Seoul National University, Seoul, Republic of Korea). The collection of ‘*UZB-GJG-1999–51*’ and ‘*C00562*’ peppers was permitted by Plant Genomics and Breeding Institute and complies with relevant institutional, national, and international guidelines and legislation. A total of 112 F_2_ pepper fruits obtained by crossing ‘*UZB-GJG-1999–51*’ and ‘*C00562*’ pepper were harvested 45–50 days after full bloom in a greenhouse at Seoul National University (Suwon, Republic of Korea). Immediately after harvest, they were precooled at 18 °C for 8 h, and then the pepper fruits were transferred to 2 °C for chilling treatment. After chilling treatment for 3 weeks, each individual fruit was cut lengthwise into half and seed browning was observed and photographed. The seed browning rate of each fruit was calculated using the following equation^20^:

$$Seed \;browning\; rate \left( \% \right) = \frac{the \;number\;of\;browned\;seeds}{{the \;number\;of\;total\; \left( {normal + browned} \right)\, seeds}} \times 100$$

**Figure 1:**
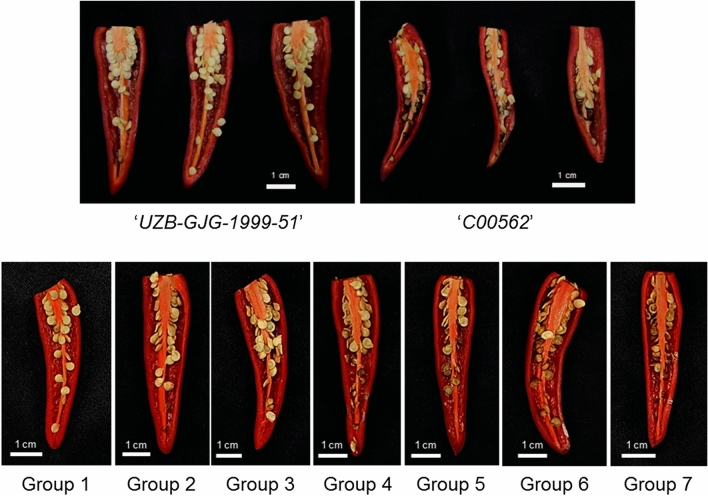
Seed browning appearances of ‘*UZB-GJG-1999–51*’, ‘*C00562*’ (upper pictures), and F_2_ groups (bottom pictures) after chilling at 2 °C for 3 weeks. The groups were designated as Group 1 through Group 7, with seed browning rates being 0–10%, 10–20%, 20–30%, 30–40%, 40–50%, 50–60%, and > 60%, respectively. The pictures in the upper panel were published as “Jasmonic acid and ERF family genes are involved in chilling sensitivity and seed browning of pepper fruit after harvest” by J. G. Lee, G. Yi, J. Seo, B. C. Kang, J. H. Choi, and E. J. Lee, 2020, in Scientific Reports, volume 10, Results section, Fig. 1. CC BY.

For further analysis, we grouped at 10% intervals using the seed browning rate for F_2_ fruits. The groups were designated as Group 1 through Group 7, with seed browning rates being 0–10%, 10–20%, 20–30%, 30–40%, 40–50%, 50–60%, and more than 60%, respectively (Fig. 1 and Supplementary Table 1). In addition, both ‘*UZB-GJG-1999–51*’ and ‘*C00562*’ fruits were treated at a chilling temperature of 2 °C for 3 weeks. All the seeds of each fruit were collected without placenta, immediately frozen in liquid nitrogen, and stored at − 80 °C for all experiments.

### Hydrogen peroxide content analysis

The hydrogen peroxide content was analyzed following a previously described method^26^ with slight modifications. First, frozen pepper seeds were completely ground into a fine powder using a mortar and pestle in liquid nitrogen. Then, 100 mg of frozen pepper seed powder was combined with 1 mL cold (4 °C) trichloroacetic acid (0.1%, w/v), and incubated at 4 °C for 10 min. All samples were centrifuged at 12,000 × *g* at 4 °C for 20 min. Each 0.5 mL supernatant was mixed with 0.5 mL of 1 M KI and 0.25 mL of 10 mM potassium phosphate buffer (pH 7.0). The mixtures were incubated in the dark at 22 °C for 20 min. Absorbance was measured at 390 nm using a microplate spectrophotometer (Biotek Epoch, Winooski, VT, USA). A standard curve was plotted using 0, 3.675, 7.35, 14.7, and 29.4 mM of hydrogen peroxide solutions.

### Total phenolic content and total antioxidant activity analysis

First, 200 mg of frozen pepper seed powder was combined with 10 mL of 80% (v/v) methanol, sonicated for 20 min, and centrifuged at 3,000 × *g* at 22 °C for 20 min. Then, each supernatant was transferred to a new 15 mL tube for total phenolic content and total antioxidant activity assays.

Total phenolic content was determined using the Folin–Ciocalteu reagent^27^. First, 50 µL of diluted solution and 50 μL Folin–Ciocalteu reagent were added to 450 μL of distilled water. The mixture was vortexed briefly and incubated at 22 °C for 5 min. Then, 150 μL of 20% (w/v) Na_2_CO_3_ and 200 μL distilled water were added, and the mixture was incubated in the dark at 22 °C for 30 min. Absorbance was measured at 750 nm using a microplate spectrophotometer (Biotek Epoch). A standard curve was plotted using 0, 12.5, 25, 50, 100, and 200 mg L^−1^ gallic acid solutions.

Total antioxidant activity was determined by evaluating the ABTS•^+^ scavenging activity^28^. The ABTS•^+^ solution was diluted with distilled water to obtain an OD_734_ = 0.7. Then, 1.2 mL diluted ABTS•^+^ solution was mixed with 10 μL of the sample. The mixture was vortexed briefly and incubated in the dark at 22 °C for 15 min. Absorbance was measured at 734 nm using a microplate spectrophotometer (Biotek Epoch). A standard curve was plotted using 0, 0.1, 0.2, 0.4, 0.8, and 1.6 mM Trolox solutions.

### Free amino acid analysis by high-performance liquid chromatography (HPLC)

Free amino acids were analyzed following a previously described method^29^ with some modifications. First, 1.2 mL of 5% trichloroacetic acid was added to 100 mg of frozen pepper seed powder, and the mixture was sonicated at 22 °C for 30 min. After centrifugation at 12,000 × *g* at 4 °C for 20 min, 1 mL of the supernatant was collected and filtered through a 0.45 μm polyvinylidene fluoride membrane filter. After mixing 5 μL of 0.4 N borate buffer (pH 10.2) and 1 μL of sample, 1 μL of o-phthalaldehyde and 1 μL of fluorenylmethyloxycarbonyl were added for derivatization. Finally, 64 μL of distilled water was added, and the mixture was analyzed by HPLC. The column was equipped with a Zorbax Eclipse AAA (4.6 × 150 mm, Agilent, Santa Clara, CA, USA), and the flow rate was set to 2 mL·min-^1^. Mobile phase A was set to 40 mM NaH_2_PO_4_ (pH 7.8), and B was set to acetonitrile:methanol:H_2_O (45:45:10, v:v:v).

### Fatty acid analysis by gas chromatography (GC)

Fatty acids were analyzed following a previously described method^30^ with some modifications. First, 100 mg of ground frozen pepper seed was placed in a Teflon-cap tube and extracted with 2 mL of methylation mixture (methanol:benzene:2,2′-dimethoxypropane:H_2_SO_4_ = 39:20:5:2, v:v:v:v) and 1 mL of heptane at 80 °C for 2 h. After cooling to 22 °C, the supernatant was collected and analyzed through GC using the Agilent 7890A system (Agilent). The column was DB-23 (0.25 × 60 × 0.25 μm, Agilent). The flame ionization detector (FID) was set at 280 °C, and the flow rates were 35 mL·min^−1^ for H_2_, 350 mL·min^−1^ for air, and 35 mL·min^−1^ for He. The injector temperature was 250 °C. The oven temperature was increased from 50 °C to 130 °C at 15 °C·min^−1^, 170 °C by 8 °C·min^−1^, and 215 °C by 2 °C·min^−1^.

### Total RNA extraction and cDNA synthesis

Frozen seeds were ground into a fine powder using a mortar and pestle in liquid nitrogen, and 100 mg powder was used for total RNA extraction using the Ribospin Seed/Fruit Kit (Geneall, Seoul, South Korea) following the manufacturer’s instructions. The extracted total RNA was used for cDNA synthesis. cDNA was synthesized using an amfiRivert Platinum cDNA Systhesis Master Mix Kit (Gendepot, Baker, TX, USA) following the manufacturer’s instructions.

### Quantitative PCR (qPCR) analysis

The cDNA was diluted to 50 ng μL^−1^, and qPCR was performed using the 2 × Real-Time PCR Master Mix (Biofact, Daejeon, South Korea) in a final volume of 20 μL and CFX Connect Real-Time System (Bio-rad, Hercules, CA, USA) under the following conditions: 95 °C for 15 min, followed by 40 cycles at 95 °C for 20 s, 55 °C for 40 s, and 72 °C for 20 s. Relative expression was determined by normalization against the expression of the pepper *Actin7*. The primers used for qPCR are listed in Supplementary Table 2 and were designed based on the reference gene set using Primer 3 plus (http://www.bioinformatics.nl/cgi-bin/primer3plus/primer3plus.cgi). Relative gene expression was calculated using the 2^−ΔΔCt^ method^31^.

### Statistical analysis

The experiments were conducted in a randomized design with three replicates of ‘*UZB-GJG-1999–51*’ and ‘*C00562*’ and five replicates of F_2_ groups. Statistical comparisons between the means of the experimental groups were performed using SPSS ver. 26.0 (IBM Corp., Armonk, NY, USA). One-way analysis of variance with Duncan’s multiple range test was performed to determine significant differences. The metabolite data were auto-scaled and used for heat-map analysis and Pearson’s correlation analysis in MetaboAnalyst 5.0 (www.metaboanalyst.ca) and correlation network analysis in Cytoscape v3.6.1 (http://cytoscape.github.io/).

## Results

### Seed browning rates of various pepper genotypes

After chilling at 2 °C for 3 weeks, there were significant differences in the seed browning rate between each F_2_ pepper fruit. We screened 112 F_2_ individuals, as well as ‘*UZB-GJG-1999–51*’ and ‘*C00562*’ pepper fruit before and after chilling treatment (Fig. 1 and Supplementary Table 1). The seed browning rate of both ‘*UZB-GJG-1999–51*’ and ‘*C00562*’ was 0% before chilling treatment and 0% and 62.99%, respectively, after chilling treatment. The seed browning rate of the F_2_ individuals ranged from 0% to 77.42%, and the average seed browning rate of groups 1 to 7 was 2.75%, 14.49%, 26.14%, 35.13%, 44.57%, 53.37%, and 66.61%, respectively.

### Hydrogen peroxide, total phenolic content, and total antioxidant activity

**Figure 2:**
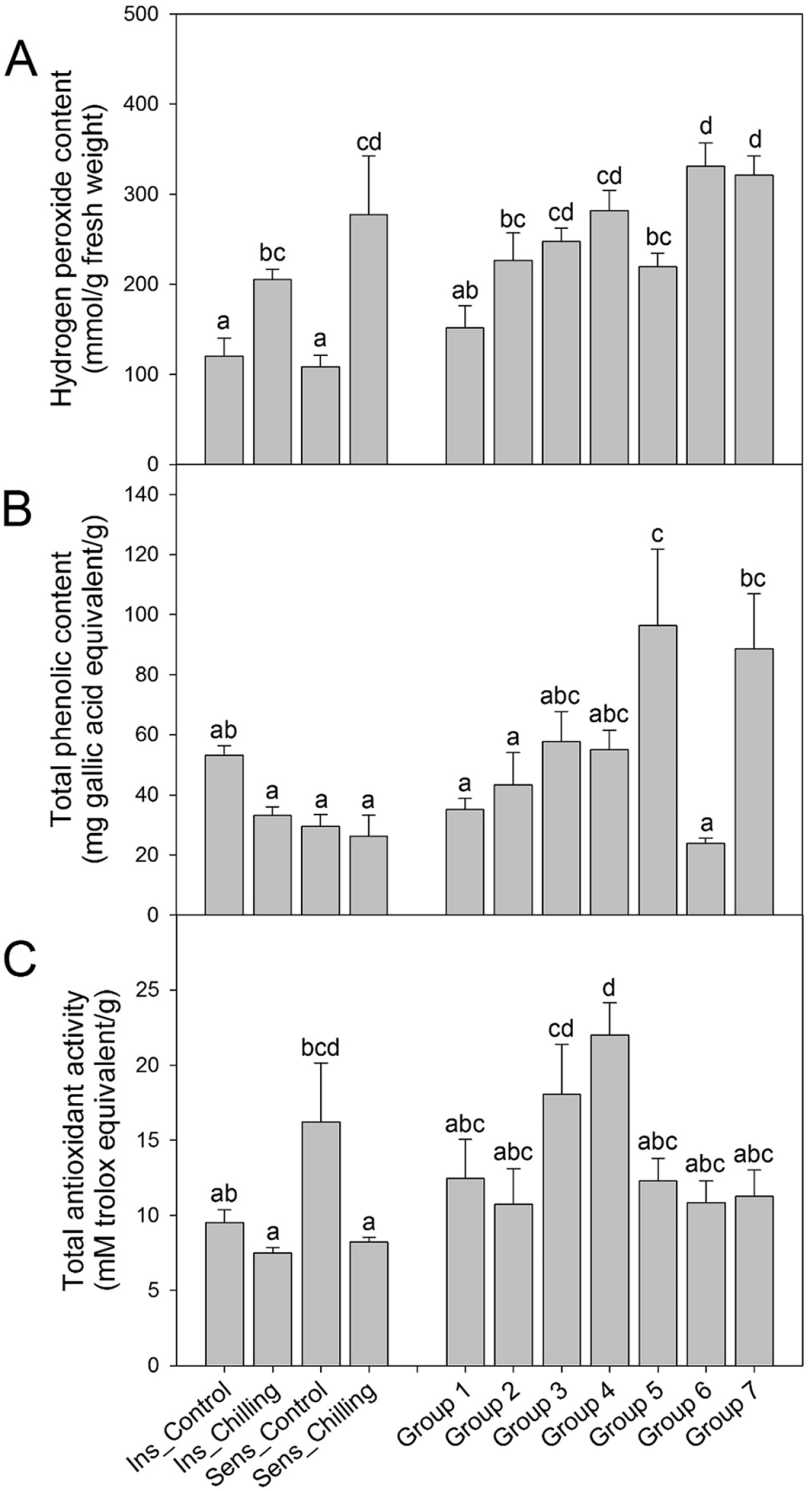
Changes in physiological traits of ‘*UZB-GJG-1999–51*’, ‘*C00562*’, and F_2_ groups after chilling treatment at 2 °C for 3 weeks. (**A**) Hydrogen peroxide content, (**B**) total phenolic content, and (**C**) total antioxidant activity. Ins, chilling-insensitive ‘*UZB-GJG-1999–51*’ pepper; Sens, chilling-sensitive ‘*C00562*’ pepper. The groups were divided from Group 1 to Group 7, and each seed browning rate was 0–10%, 10–20%, 20–30%, 30–40%, 40–50%, 50–60%, and > 60%, respectively. Vertical bars are means ± SE. Different letters represent significant differences (*p* < 0.05) in Duncan’s multiple range test.

The changes in hydrogen peroxide content, total phenolic content, and total antioxidant activity were confirmed according to the seed browning rate (Fig. 2). Hydrogen peroxide content increased in both ‘*UZB-GJG-1999–51*’ and ‘*C00562*’ peppers after chilling at 2 °C. In addition, as the seed browning rate increased in the F_2_ individuals, the hydrogen peroxide content significantly increased in groups 6 and 7 when compared to groups 1 and 2 (Fig. 2A). There was no significant difference in total phenolic content before and after chilling in both ‘*UZB-GJG-1999–51*’ and ‘*C00562*’ peppers. Moreover, F_2_ individuals showed significantly higher content in groups 5 and 7, but no significant difference was found in the other groups (Fig. 2B). Before or after chilling treatment, total antioxidant activity did not differ in ‘*UZB-GJG-1999–51*’ pepper, but was significantly decreased in ‘*C00562*’ pepper after chilling treatment. In F_2_ individuals, group 4 showed significantly higher activity, but there was no significant difference between the other groups (Fig. 2C).

### Heat-map analysis of free amino acid and fatty acid content

**Figure 3:**
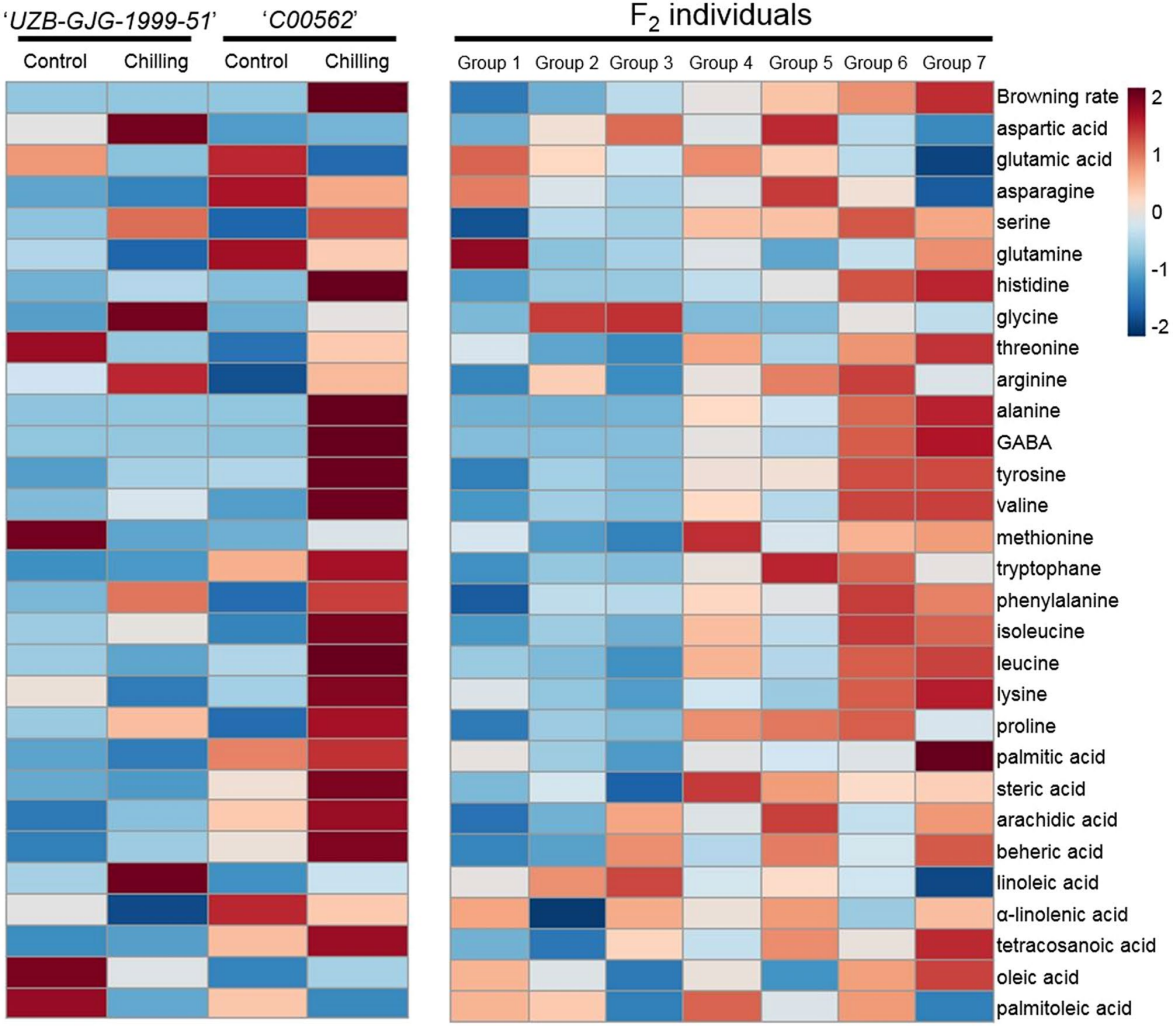
Heat map of free amino acids and fatty acids in ‘*UZB-GJG-1999–51*’, ‘*C00562*’, and F_2_ groups after chilling treatment at 2 °C for 3 weeks. The groups were divided from Group 1 to Group 7, and each seed browning rate was 0–10%, 10–20%, 20–30%, 30–40%, 40–50%, 50–60%, and > 60%, respectively. The mean fold-change magnitude for overall metabolites in each group is color-coded. Increases are shown in red while blue indicates decreases. This figure was created with freely available MetaboAnalyst (version 5.0, https://www.metaboanalyst.ca/).

The content of 20 amino acids and nine fatty acids was quantified by HPLC and GC-FID. To confirm the relationships between the seed browning rate and metabolites, a heat-map analysis was performed using changes in metabolites (Fig. 3). In ‘*UZB-GJG-1999–51*’ pepper, fatty acid and amino acid content did not show an obvious change. These results might be because the seeds were less damaged by chilling treatment, and as a result, seed browning did not occur. Valine, isoleucine, and linoleic acid content slightly increased, and palmitic acid content slightly decreased. Conversely, in '*C00562*' pepper, in which seed browning occurred following chilling treatment, the content of most amino acids, except for glutamic acid, increased. In addition, palmitic acid content increased, and linoleic acid content decreased. In F_2_ individuals, as the seed browning rate increased, the content of a number of amino acids, including branched-chain amino acids (BCAAs), GABA, and phenylalanine, tended to increase. However, aspartic acid, glutamic acid, asparagine, and glycine were not significantly correlated with the seed browning rate. In the case of fatty acids, palmitic acid, a saturated fatty acid, increased as the seed browning rate increased, whereas linoleic acid, an unsaturated fatty acid, decreased.

### Relative gene expression analysis by qPCR

**Figure 4:**
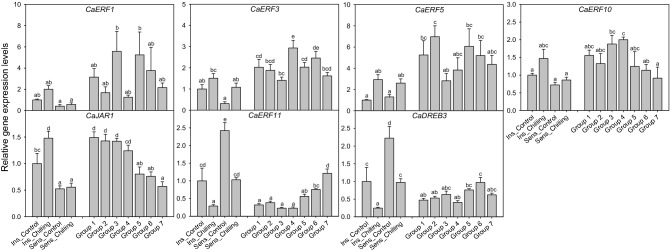
Relative expression levels of *CaJAR1* and *CaERF* family genes in ‘*UZB-GJG-1999–51*’, ‘*C00562*’, and F_2_ groups after chilling treatment at 2 °C for 3 weeks. Ins, chilling-insensitive ‘*UZB-GJG-1999–51*’ pepper; Sens, chilling-sensitive ‘*C00562*’ pepper. The groups were designated as Group 1 through Group 7, with seed browning rates being 0–10%, 10–20%, 20–30%, 30–40%, 40–50%, 50–60%, and > 60%, respectively. Vertical bars are means ± SE. Different letters represent significant differences (*p* < 0.05) in Duncan’s multiple range test.

From the gene expression analysis of *CaERF 1*, *3*, *5*, and *10*, selected as positive regulator candidate genes for chilling response in previous studies, there was no significant difference in relative gene expression levels before and after chilling in both '*UZB-GJG-1999–51*' and '*C00562*' peppers. Moreover, expression levels of *CaERF 1*, *3*, *5*, and *10* tended to be higher in '*UZB-GJG-1999–51*' than in '*C00562*', but there was no significant difference between the genotypes (Fig. 4). In the analysis of F_2_ individuals, *CaERF1* expression levels were higher in groups 3 and 5 but not significantly. *CaERF3* expression level was significantly higher in group 4 than in other groups except group 6. *CaERF5* expression level was significantly higher in group 2 than in group 3. In addition, *CaERF10* expression levels were significantly higher in groups 3 and 4 than in group 7, but there was no tendency to change according to the seed browning rate. However, in the case of *CaJAR1*, another candidate positive regulator gene, the expression level was significantly increased after chilling in '*UZB-GJG-1999–51*’ pepper. In addition, from the analysis between F_2_ individuals, it was confirmed that the expression level significantly decreased as the seed browning rate increased.

*CaERF11* and *CaDREB3*, candidates for negative regulator genes in chilling response, showed similar gene expression trends. In both '*UZB-GJG-1999–51*' and '*C00562*' pepper, the expression level of *CaERF11* and *CaDREB3* significantly decreased after chilling treatment, and was higher in '*C00562*' than in ‘*UZB-GJG-1999–51*’. In the analysis of F_2_ individuals, the expression level of *CaERF11* increased significantly with the increase in seed browning rate, but *CaDREB3* was significantly higher only in group 6, and there was no significant difference in the other groups.

### Correlations between seed browning rate and analysis factors

**Figure 5:**
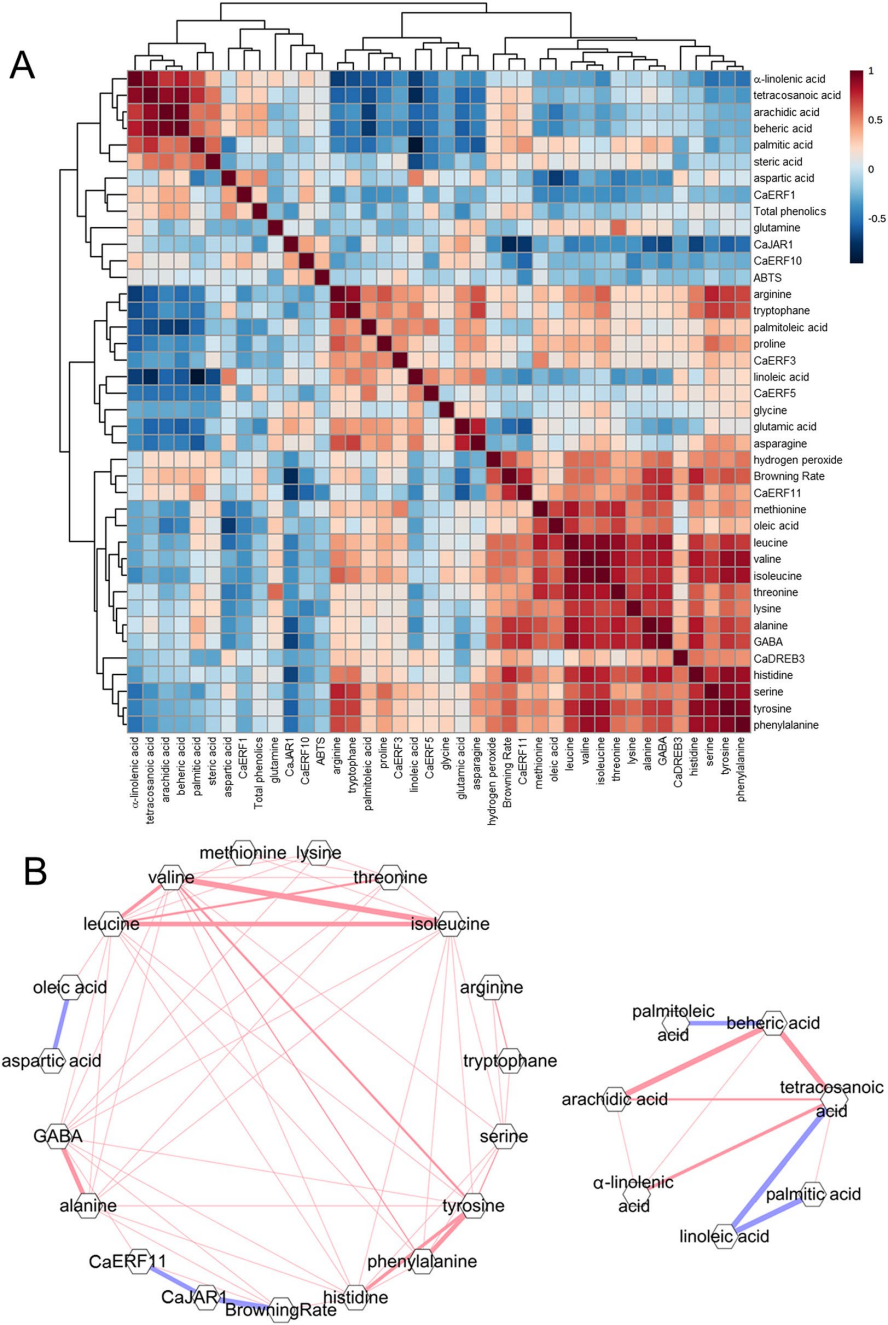
Correlation heat map (**A**) and networks (**B**) among physiological traits, metabolic compounds, and expression levels of *CaJAR1* and *CaERF* family genes in ‘*UZB-GJG-1999–51*’, ‘*C00562*’, and F_2_ groups after chilling treatment at 2 °C for 3 weeks. Positive and negative correlations are shown as red and blue lines, respectively, with the line thickness corresponding to a higher or lower Pearson’s correlation for correlation ≥|0.70|. Correlation heat map is created with freely available MetaboAnalyst (version 5.0, https://www.metaboanalyst.ca/) and networks are created with Cytoscape software (version3.6.1, https://cytoscape.github.io/).

To identify the factors involved in pepper seed browning, correlation analysis was performed using physiological traits, metabolites, and gene expression levels. First, the results of Pearson's correlation heat-map analysis confirmed that seed browning was positively correlated with *CaERF11* expression level and hydrogen peroxide content at r = 0.7622 and 0.6607, respectively (Fig. 5A). In contrast, it was confirmed that seed browning was negatively correlated with *CaJAR1* expression level (r =  − 0.7996). Furthermore, *CaJAR1* was positively correlated with *CaERF10* expression level and total antioxidant activity. In addition, *CaERF1* expression level was positively correlated with aspartic acid content and total phenolic content, and *CaERF3* and *CaERF5* expression levels were positively correlated with linoleic acid content.

To clearly visualize the correlation between seed browning and the analyzed factors, a correlation network was analyzed based on Pearson's correlation coefficient ( ≥|0.7|, *p* < 0.05) (Fig. 5B). Through correlation network analysis, it was confirmed that *CaJAR1* expression level was negatively correlated with the seed browning rate and *CaERF11* expression level (r =  − 0.7996 and − 0.7254, respectively). In addition, isoleucine, leucine, and valine, belonging to BCAA, were positively correlated with each other. In terms of fatty acids, oleic acid was negatively correlated with aspartic acid (r =  − 0.7158), and palmitic acid and linoleic acid, and the representative saturated and unsaturated fatty acids, respectively, were negatively correlated (r =  − 0.9530).

**Figure 6:**
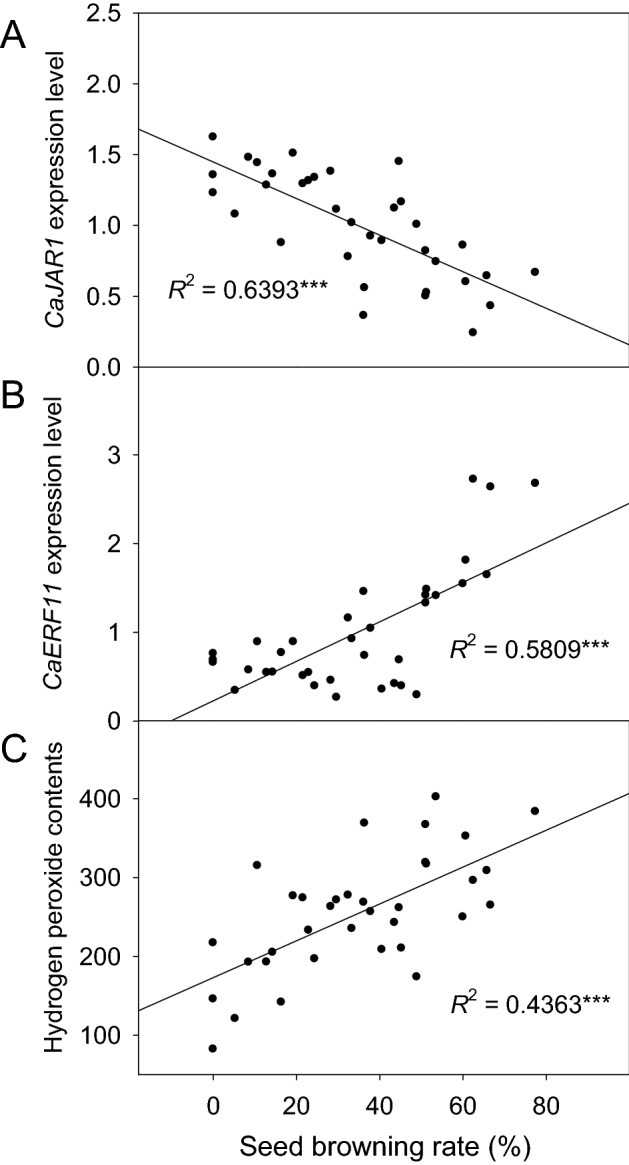
Correlation analysis of the seed browning rate with *CaJAR1* expression level (**A**), *CaERF11* expression level (**B**), and hydrogen peroxide content (**C**) in ‘*UZB-GJG-1999–51*’, ‘*C00562*’, and F_2_ groups after chilling treatment at 2 °C for 3 weeks.

*CaJAR1* and *CaERF11* expression levels and hydrogen peroxide content, which were correlated with the seed browning rate in the heat-map and network analyses, were used to confirm the individual correlation with the seed browning rate (Fig. 6). *CaJAR1* expression level was confirmed as the coefficient of determination (*R*^*2*^) with a seed browning rate of 0.6393 (*p* < 0.001) (Fig. 6A). The *R*^2^ for seed browning with *CaERF11* expression level and hydrogen peroxide content were 0.5809 and 0.4365, respectively (Fig. 6B,C).

## Discussion

As a result of chilling, in 112 F_2_ individual pepper fruits obtained from crossing chilling-insensitive *'UZB-GJG-1999–52*' and chilling-sensitive '*C00562*' peppers, the seed browning rate was widely distributed, ranging from 0 to 77.42% (Fig. 1). From these results, we inferred that the seed browning rate and chilling sensitivity of pepper are affected by genetic factors. To identify the factors influencing chilling sensitivity, F_2_ individuals were classified and grouped based on the seed browning rate, and physiological traits, metabolites, and gene expression levels were identified.

First, it was confirmed that the hydrogen peroxide content was significantly correlated with the browning rate (r = 0.6607) and increased as the seed browning rate increased (Fig. 2A). Furthermore, previous studies reported that total phenol content and phenylalanine increased, showing a significant correlation with tissue browning during chilling stress^32^, while total phenol content was reported to decrease in pepper seeds during chilling treatment^33^. In the present study, total phenol content was not significantly correlated with seed browning after chilling (Fig. 2B). However, phenylalanine content was positively correlated with the seed browning rate (r = 0.6120) (Figs. 3 and 5).

After chilling treatment, total antioxidant activity was not significantly different between the groups, except for group 4 (Fig. 2). Also, in this study, the tendency of changes in the antioxidant activity and total phenol content associated with chilling sensitivity was not similar, and there was no significant difference. These results are not consistent with those of a previous study wherein changes in the antioxidant activity were observed to have a high correlation with the changes in the total phenol content^34^. Under abiotic stress, such as chilling, photochemical systems I and II are damaged, and the generated excitation energy is transferred to oxygen molecules to generate harmful ROS, such as hydrogen peroxide, O_2_^−^, and OH^−^^35^. Plants have enzymatic and non-enzymatic antioxidant systems against ROS. Antioxidant enzymes include superoxide dismutase, catalase, ascorbate peroxidase, and glutathione-S-transferase^36^, and non-enzymatic antioxidant metabolites include ascorbic acid, glutathione, and phenolic compounds^37^. Total phenol content and antioxidant activity vary mainly based on genotypes and environmental factors, such as storage temperature and duration^38,39^. Even in previous studies, the changes in the total phenol content and antioxidant capacity were not consistent depending on the storage period^38,40^, probably because of various factors, such as genetics, ROS, and expression of related genes.

In the present study, palmitic acid, linoleic acid, GABA, leucine, valine, and isoleucine had a significant correlation with seed browning (Fig. 3). Palmitic acid and linoleic acid, which are saturated and unsaturated fatty acids, respectively, were negatively correlated with r =  − 0.9530 (Fig. 5). When plants are subjected to chilling stress, lipid peroxidation occurs, and unsaturated fatty acids are oxidized to saturated fatty acids. ROS, generated during this process, promotes chilling response signaling but accumulates cell damage within the plant^41^. Therefore, changes in the content of palmitic acid and linoleic acid in pepper seeds after chilling treatment serve as indicators of damage and ROS generation induced by chilling stress.

After chilling treatment, the change in GABA content was positively correlated with the seed browning rate (r = 0.7402) (Figs. 3 and 5). GABA is involved in most abiotic stresses and is synthesized upon recognition of Ca^2+^ ions generated by damage in plants^42^. Therefore, it is expected that the GABA content will increase as the chilling stress damage increases.

Changes in the content of isoleucine, leucine, and valine were found to have correlation coefficients (r) of 0.5099, 0.5688, and 0.5674, respectively, with the seed browning rate after chilling treatment. In addition, it can be seen that the correlation coefficient between each of the three amino acids was high from 0.8120 to 0.9595 (Fig. 5). These amino acids belong to BCAA, and they share a biosynthetic pathway with pyruvic acid^43^. In particular, isoleucine plays an important role in activating JA signaling by forming JA-Ile through JAR1^44^. Previous studies have confirmed that the endogenous JA content of pepper fruits and seeds is low, and the content of isoleucine increases with increasing seed browning^11^. Therefore, we inferred that the chilling response mechanism of pepper under chilling stress is regulated according to the expression level of *CaJAR1*, a gene that synthesizes JA-Ile. In the present study, the expression level of *CaJAR1* was negatively correlated with the seed browning rate (r =  − 0.7996), and the expression level showed a tendency to decrease as the seed browning rate increased after chilling treatment (Figs. 4 and 6A). These results suggest that *CaJAR1* is a positive regulator that influences the chilling sensitivity of pepper.

In the case of *CaERF1*, *3*, *5*, and *10*, contrary to the expectation, there was no significant correlation with the seed browning rate after chilling (Figs. 4 and 5). In a previous study, the expression levels of *CaERF 1*, *3*, *5*, and *10* significantly increased in the short-term chilling treatment for 24 h, and were significantly higher in chilling-insensitive '*UZB-GJG-1999–51*' pepper. However, there was no significant difference in seed browning after 3 weeks of chilling. According to previous research, in the case of the *ERF*, the expression level was high at the beginning of the chilling treatment, and decreased thereafter^45^. In addition, because the *ERF* family genes are representative abiotic stress response genes, it is expected that the expression of *ERFs* would not be directly induced by chilling stress, but rather by damage, such as ROS generated by chilling stress, resulting in different results from previous studies.

In the case of *CaERF11* and *CaDREB3*, which are candidate genes for negative regulators of pepper chilling response, the expression level of *CaDREB3* after chilling was not significantly correlated with the seed browning rate (Fig. 4). However, the expression level of *CaERF11* was highly correlated with the seed browning rate (r = 0.7622), and it increased as the seed browning rate increased. Studies have shown that *ERF11* promotes stem growth through the activation of gibberellin^46^ and suppresses the abiotic stress response of plants through antagonism with *ERF6*^47^. We suggest that *CaERF11* is a negative regulator of the chilling response in pepper.

In conclusion, after chilling treatment, cell membrane peroxidation occurs, and ROS, including hydrogen peroxide, accumulate in the cells, and finally, seed browning of pepper occurs. In chilling-sensitive peppers, this process proceeds quickly and unlike in chilling-insensitive peppers, it proceeds slowly and weakly. Factors influencing the chilling sensitivity of pepper are expected to be regulated by the expression level of *CaJAR1*, which is involved in JA signaling activation, and the expression level of *CaERF11*, which inhibits abiotic stress response in plants.

## Supplementary Information

Supplementary Information.

## Data Availability

The original contributions presented in the study are included in the article/Supplementary Material; further inquiries can be directed to the corresponding author.
