# Supplementary material for: Jasmonate resistant 1 and ethylene responsive factor 11 are involved in chilling sensitivity in pepper fruit (Capsicum annuum L.)
Source: Sci Rep. 2022 Feb 24;12:3141. doi: 10.1038/s41598-022-07268-3 (PMC8873250; doi:10.1038/s41598-022-07268-3)
Supplement: Supplementary file 1 — Supplementary Information. [file 41598_2022_7268_MOESM1_ESM.docx]

Supplementary Info

**Supplementary Table S1**. Seed browning rates of ‘*UZB-GJG-1999-51*’, ‘*C00562*’ peppers, and F2 groups after chilling treatment.

| **Group** | **Browning rate (%)** |
| --- | --- |
| *UZB-GJG-1999-51*_0 d | 0 |
|  | 0 |
|  | 0 |
| *UZB-GJG-1999-51*_21 d | 0 |
|  | 0 |
|  | 0 |
| *C00562*_0 d | 0 |
|  | 0 |
|  | 0 |
| *C00562*_21 d | 62.96 |
|  | 71.87 |
|  | 54.16 |
| Group 1 | 0 |
|  | 0 |
|  | 0 |
|  | 5.26 |
|  | 8.51 |
| Group 2 | 10.64 |
|  | 12.82 |
|  | 14.29 |
|  | 16.36 |
|  | 18.37 |
| Group 3 | 22.92 |
|  | 24.39 |
|  | 25.53 |
|  | 28.26 |
|  | 29.63 |
| Group 4 | 32.43 |
|  | 33.33 |
|  | 35.71 |
|  | 36.36 |
|  | 37.84 |
| Group 5 | 40.54 |
|  | 43.55 |
|  | 44.68 |
|  | 45.24 |
|  | 48.84 |
| Group 6 | 51.06 |
|  | 51.06 |
|  | 51.28 |
|  | 53.57 |
|  | 59.92 |
| Group 7 | 60.71 |
|  | 62.5 |
|  | 65.79 |
|  | 66.67 |
|  | 77.42 |

**Supplementary Table S2.** Primers used for qPCR analysis in the present study.

| **Gene** | **Forward primer sequence**  **(5′-3′)** | **Reverse primer sequence**  **(3′-5′)** | **Size (bp)** |
| --- | --- | --- | --- |
| *CaActin7* | TCTCCCCAACTACAAACAAC | CTGCTGTTTTAGGCAAGTCT | 160 |
| *CaERF1* | TTTAAATTTCCCGAGCCAGA | ATTTCTCAGCTACACCGTTT | 143 |
| *CaERF3* | CGATCAACTCTAGCTTCCAA | GTCTCAAATGTTCCTAGCCA | 143 |
| *CaERF5* | ACCGAGGTCCAAATATCATG | GGGATTGTGGAAATGCAAAA | 148 |
| *CaERF10* | TTCTTGTGGATGTTCGTGAT | CCAGAGTTGTTAGTTGGTGA | 141 |
| *CaERF11* | CCATTTGACAAGTCCATCTT | ATGCCAGTGGTAGAAGAAAA | 142 |
| *CaDREB3* | AGGTCACGTAGCCTTACTCA | AACAGGGCACTTCCATACTA | 146 |
| *CaJAR1* | CTTGGCTGTAGCAAAACACT | GAATCTTCCCTCATCAGACA | 148 |
